# Serine synthesis and transport mediate the synergistic and detoxifying effects of lienal peptides on cisplatin

**DOI:** 10.3389/fphar.2025.1646217

**Published:** 2026-01-16

**Authors:** Ke Ding, Tengjie Yu, Xiaowei Zhang, Kangrui Hu, Shuying Mao, Tingting Zhang, Ye Liu, Zhongbo Wang, Xin Fan, Wei Liu, Dianzhui Zhang, Laipeng He, Lin Xie, Guangji Wang, Yan Liang

**Affiliations:** 1 Jiangsu Provincial Key Laboratory of Drug Metabolism and Pharmacokinetics, Key Laboratory of Natural Medicines, China Pharmaceutical University, Nanjing, China; 2 Nutrition Department, Hebei General Hospital, Shijiazhuang, Hebei, China; 3 Hebei Zhitong Biopharmaceutical Co., Ltd., Gucheng, Hebei, China

**Keywords:** cisplatin, lienal peptides, metabolome, serine, SFXN1

## Abstract

Lienal peptides (LPs), one kind of animal-derived traditional Chinese medicine, are used clinically as an adjunctive therapeutic drug in oncology. Cisplatin (DDP) is a broad-spectrum chemotherapy drug widely used for non-small cell lung cancer (NSCLC) but is limited by a series of toxic side effects. To date, the feasibility of using LPs and DDP in combination remains unclear. The present study aimed to explore the feasibility of LPs combined with DDP as an adjuvant therapy for NSCLC by examining the effects of LPs on the efficacy and toxicity of DDP. Our findings demonstrated that LPs exhibited significant antitumor activity in Lewis lung carcinoma (LLC)-bearing mice when administered either alone or combined with DDP. Moreover, LPs treatment notably improved survival rate, alleviated renal and hepatic impairment, and reversed DDP-induced metabolic disorder. Additionally, our data indicated that tumor growth led to dysregulated serine metabolism. LPs could significantly normalize serine levels by modulaitng the synthesis of serine in gut microbiota and its SFXN1-mediated transport in tumor cells. Thus, this study for the first time reveals the auxiliary anti-tumor effect of LPs from the perspective of amino acid metabolism, supporting LPs as a promising adjuvant to DDP in NSCLC.

## Graphical Abstract

**Figure:**
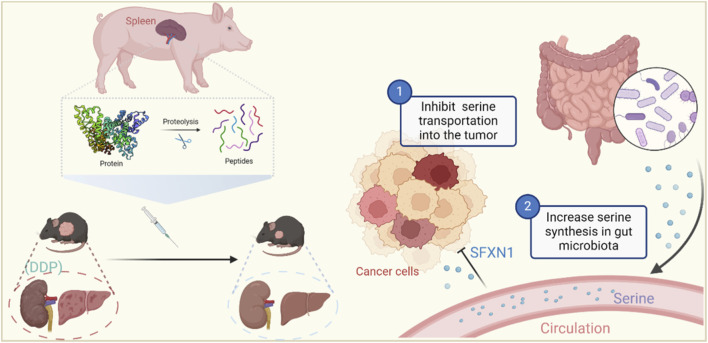

## 1 Introduction

Non-small cell lung cancer (NSCLC) accounts for 85% of all lung cancer cases and remains the leading cause of cancer-related mortality worldwide. For stage III NSCLC, chemotherapy is still the first choice, with cisplatin (DDP) being the most commonly used broad-spectrum agent (Molina et al., 2008). DDP forms intra- and inter- DNA crosslinks that impair DNA replication and repair, ultimately triggering cancer cell apoptosis. The prominent nephrotoxicity of DDP was mainly manifested as damage to renal tubular epithelial cells, liver damage, bone marrow suppression and drug allergies in some patients (Pignon et al., 2008). To improve the efficacy of DDP and alleviate its adverse reactions, DDP is often combined with other therapies in NSCLC, including immune checkpoint blockers (Fournel et al., 2019) and molecular targeted inhibitors (Wang et al., 2021), etc. However, these adjuvant strategies still require additional clinical trials and long-term survival data (Saw et al., 2021). Certain traditional Chinese medicines (TCMs), such as magnolols and Poria cocos, have also been reported to reduce the liver and kidney toxicity of DDP (Zou et al., 2021; Li et al., 2022; Mao et al., 2022). For instance, Li et al. (2020) found that honokiol could attenuate DDP-induced renal injury by regulating fatty acid oxidation through Sirt3-mediated deacetylation of liver kinase B1 and activation of AMP-activated protein kinase. Nonetheless, the feasibility of combining these TCM-derived ingredients with DDP remains to be validated.

Protein hydrolysates are complex mixtures extracted from animal or plant proteins through enzymatic hydrolysis. Their main components are peptides, oligopeptides, amino acids, and nucleic acids and may also contain carbohydrates, trace mineral elements, and other organic compounds (Schaafsma, 2009; Calvo et al., 2014; Colla et al., 2015). Notably, compared with intact proteins, hydrolysates often exhibit superior solubility, digestibility, and bioactivity. It has been reported that protein hydrolysates have anti-aging (Zhu et al., 2021), immunomodulation (Liu et al., 2017; Lozano-Ojalvo et al., 2019; Yang et al., 2019; Martinez-Blanco et al., 2020; Zhang et al., 2021a), neuroprotection (Chataigner et al., 2021; Myagmar et al., 2022), antioxidation (Deng et al., 2020), and other effects.

Lienal peptides (LPs) are extracted from porcine or bovine spleen via tryptic digestion and downstream processing. The main activity of LPs lies in immune regulation. Numerous literatures have shown that LPs could improve the immunosuppression of cyclophosphamide-treated Lewis lung carcinoma (LLC)-bearing mice, rebalance CD4^+^ and CD8^+^ T cells, enhance NK cell cytotoxicity (Rao et al., 2018; Wu et al., 2018), and inhibit the overexpression of inflammatory factors in YAC-1 cells through NF-κB pathway (Wang et al., 2018). Clinically, LPs for injection has been widely used in adjuvant tumor chemotherapy due to its safety and broad immunomodulatory effects (Huang et al., 2015). Even so, the safety and efficacy of combing LPs with chemotherapy in the treatment of cancer still require preclinical and clinical validation.

Our previous research devoted to developing a robust qualitative and quantitative analysis platform for mapping protein hydrolysate profiles and pharmacokinetics, and a total of main 247 unique peptides corresponding to 55 proteins in LP oral liquids were identified based on NanoLC-Orbitrap-MS/MS system. Among them, 14 peptides, including ADFTEISK, AGGTTVTQGVETTKPS, AVGHLDDLPGA, DIPGLTDTTVPR, DNIQGITKP, DNIQGITKPA, GFGGVQVSPPNENI, GIITNWDDMEK, GIVTNWDDMEK, LTEAPLNPK, SGGTTMYPGIAD, SYELPDGQV, TEAPLNPK, and VNVDEVGGEALG showed signal intensity > 10^4^ (Mao et al., 2023). Based on this foundation, our present study investigated the feasibility of LPs combined with DDP as an adjuvant therapy for NSCLC by detecting the impact of LPs on DDP’s pharmacological intensity and toxicity. We found that the administration of LPs notably improved survival rate, alleviated renal and hepatic impairment, and reversed DDP-induced disorder of metabolic profiles. Mechanistically, given that rapidly proliferating tumors scavenge exogenous nutrients and exhibit pervasive metabolic reprogramming (van der Meij et al., 2019; Vettore et al., 2020), with significant alterations in amino acid levels within the tumor microenvironment (Wang and Zou, 2020; Martinez-Reyes and Chandel, 2021), we focused on serine metabolism. Serine has been reported as a key metabolite for oncogenesis, progression, and adaptive immunity. The synthesis of serine can drive the serine glycine one-carbon pathway (SGOCP), the tricarboxylic acid (TCA) cycle, and pathways for lactate and lipid synthesis. Influenced by various tumor microenvironment factors, overactivation of serine metabolism can drive malignant proliferation, epithelial-to-mesenchymal transition (EMT), metastasis, angiogenesis, and drug resistance (Shunxi et al., 2023). In this study, we innovatively elucidated that LPs could enhance the therapeutic efficacy and reduce the side effects of DDP by correcting amino acid metabolic disorders, thereby providing a rationale mechanistic framework centered on serine. Collectively, for the first time, we revealed the auxiliary anti-tumor effect of LPs from the perspective of amino acid metabolism, providing a new clinical treatment strategy for NSCLC.

## 2 Materials and methods

### 2.1 Chemicals and reagents

The oral peptide solution of LPs (125 mg/mL, calculated as protein) was generously provided by Zhitong Biopharmaceutical Co., Ltd. (Hebei, China). DDP was supplied by Haosen Pharmaceutical Co., Ltd. (Jiangsu, China). DEPC water and CCK-8 assay kit were purchased from Beyotime Institute of Biotechnology (Jiangsu, China). Ki67 antibody was obtained from Abcam Ltd. (Cambridge, United Kingdom). RNAiso Plus was from Takara Biotechnology Co., Ltd. (Shiga, Japan) and Prime Script RT Master Mix and SYBR Premix Ex Taq were from Novozymes Biological Technology Co., Ltd. (Jiangsu, China). Primers were designed and synthesized by Invitrogen (CA, United States). Ultra-pure water was prepared by the Milli-Q system from Millipore Corporation (Billerica, MA, United States). Cr colorimetric assay kit (creatinine oxidase method), urea colorimetric assay kit (urease method), AST/GOT colorimetric assay kit, and ALT/GPT colorimetric assay kit were all purchased from Elabscience Biotechnology Co., Ltd. (Wuhan, China). All other chemicals were purchased from Sigma-Aldrich (St. Louis, MO, United States).

### 2.2 Cell culture

LLC, A549 NSCLC cells, HepG2 hepatocellular carcinoma cells, LO2 liver cells, and RAW 264.7 mouse monocyte macrophage leukemia cells were purchased from American Type Culture Collection (Rockville, MD, United States). LLC and A549 cells were routinely cultured in RPMI-1640 medium, and HepG2, LO2 cells, RAW 264.7 cells were cultured in DMEM medium. Both medium contained 10% fetal bovine serum, 100 U/mL penicillin and streptomycin, and all reagents mentioned for cell culture were purchased from Gibco/Invitrogen (CA, United States). The cells were grown in an atmosphere of 5% CO_2_ at 37 °C and passaged when reaching 80% confluence.

### 2.3 Cell viability assay

Cells were added to a 96-well plate at 1× 10^4^ cells/well. After 12 h, LPs at 0.5%, 1%, or 2% (v/v) were added to the culture medium. Cell viability assay was measured 24 h later by adding CCK-8 reagent strictly according to manufacturer instructions. The absorbance at 450 nm for each well was measured using a microplate reader (BioTek, United States).

### 2.4 Animals and treatments

SPF C57BL/6J mice (male, 6 weeks old, 19–20 g) were purchased from Vital River Laboratory Animal Co., Ltd. (Beijing, China) (SCXK 2019-0001). The mice were housed at controlled temperature of 20 °C–22 °C with a 12 h light-dark cycle, 50%–60% humidity, and free access to food and water. After habituating for a week, LLC cells in suspension (1 × 10^6^ cells/125 µL PBS/mouse) were subcutaneously implanted near the right scapulae of mice. The tumor-bearing mice were then randomly assigned to different treatment groups (n = 8).

For therapeutic evaluation of LPs, 40 mice were allocated to control, model, DDP, DDP + LPs, and LPs groups. Mice in LPs and DDP + LPs groups were intragastrically administrated daily with LPs (10 mL/kg) on the second day after tumor implantation. The treatment lasted for 25 days. When the tumor volume reached 50 mm^3^, mice in DDP and DDP + LPs groups were intraperitoneal treated with DDP (2.5 mg/kg) once every 3 days until the cumulative dose reached 12.5 mg/kg. Mice in control and model groups were given the same volume of saline. Besides, in the establishment of acute DDP toxicity model, DDP was administerted at 5 mg/kg every 3 days until the cumulative dose reached 15 mg/kg. Mice were euthanized on day 8 from the first DDP dose.

### 2.5 Xenograft tumor model studies

The mice were weighed, and the (a) minor and (b) major axes were measured with a caliper every 3 days after DDP administration. Tumor volume (mm^3^) = 0.5 × a^2^ × b. At the end of experiments, mice were sacrificed and tumor xenografts were excised carefully and weighed. Tumor inhibition rate (%) = (1 - mean tumor weight in treatment group/mean tumor weight in model group) × 100%.

### 2.6 Histopathological and immunohistochemical (IHC) analysis

Fresh tumor and spleen specimens from mice were immersed in 4% paraformaldehyde for 2 days. Subsequently, the fixed tissues were sectioned and stained using a picrosirius red solution for 1 hour according to histopathological techniques. Following three washes with acidified water, tissue sections were counterstained with Carazzi’s hematoxylin.

As to IHC for Ki67, the slides of tumor tissues were deparaffinized and subjected to antigen retrieval (citrate buffer). After PBS washing, the sections were subjected to endogenous peroxidase blocking with 3% H_2_O_2_ in dark for 25 min. Subsequently, sections were blocked with 3% bovine serum albumin, and with primary antibody against mouse ki67 (1:300, Servicebio, Wuhan, China) at 4 °C overnight, followed by incubation with horse reddish peroxidase-conjugated secondary antibodies at room temperature for 50 min. Finally, the sections were stained using 3,3′-Diaminobenzidine tetrahydrochloride substrate and counterstained with hematoxylin.

### 2.7 qPCR analysis of immune checkpoint, immune factors, and serine-related enzymes/transporters

TRIzol (1 mL, Invitrogen Co., CA, United States) was added to 50 mg of mouse tumor tissues to prepare homogenate, and 200ul trichloromethane was then added to the supernatant. After centrifugation at 12,000 g × 15 min, the upper layer was transferred and 500 μL of isopropanol was added to precipitate RNA. Subsequently, 1 mL of 75% ethanol was added, and after centrifugation at 12,000 g × 5 min, the RNA precipitate was dried and dissolved in 10 μL of DEPC water. cDNA was synthesized using a High-Capacity RNA-to-cDNA Kit (Applied Biosystems, Foster City, CA, United States), and RT-PCR was carried out on a Thermal Cycler Dice TM Real-Time System (TaKaRa Code: TP800). The primer sequences for the target genes were listed in Table 1.

**TABLE 1:** Summary of primer sequence.

Primers	Forward	Reverse
PD-L1	GCT​CCA​AAG​GAC​TTG​TAC​GTG	TGA​TCT​GAA​GGG​CAG​CAT​TTC
PDCD1	ACC​CTG​GTC​ATT​CAC​TTG​GG	CAT​TTG​CTC​CCT​CTG​ACA​CTG
CD80	ACC​CCC​AAC​ATA​ACT​GAG​TCT	TTC​CAA​CCA​AGA​GAA​GCG​AGG
CD86	TCT​CCA​CGG​AAA​CAG​CAT​CT	CTT​ACG​GAA​GCA​CCC​ATG​AT
CTLA-4	GCT​TCC​TAG​ATT​ACC​CCT​TCT​GC	CGG​GCA​TGG​TTC​TGG​ATC​A
IL-10	CAA​GGA​GCA​TTT​GAA​TTC​CC	GGC​CTT​GTA​GAC​ACC​TTG​GTC
TGF-β	TGA​CGT​CAC​TGG​AGT​TGT​ACG​G	GGT​TCA​TGT​CAT​GGA​TGG​TGC
IFN-γ	CTT​TGG​ACC​CTC​TGA​CTT​GAG	TTC​CAC​ATC​TAT​GCC​ACT​TGA​G
TNF-α	CTG​TAG​CCC​ACG​TCG​TAG​C	TTGAGATCCATGCCGTTG
IL-2	TGA​GCA​GGA​TGG​AGA​ATT​ACA​GG	GTC​CAA​GTT​CAT​CTT​CTA​GGC​AC
Perforin	GGG​TTT​ATC​AGT​TGT​GCC​GTC	TAG​CAG​ATG​GAC​AGG​GGT​GTA​G
Granzyme B	GGA​ACA​CCT​CTT​CTG​CCA​CC	AGC​ATT​AGA​TAA​CAT​TCT​CGG​GG
PHGDH	ATG​GCC​TTC​GCA​AAT​CTG​C	AGT​TCA​GCT​ATC​AGC​TCC​TCC
PSAT1	AAG​CCA​CCA​AGC​AAG​TGG​TTA	GAT​GCC​GAG​TCC​TCT​GTA​GTC
PSPH	AGG​AAG​CTC​TTC​TGT​TCA​GCG	GAG​CCT​CTG​GAC​TTG​ATC​CC
SFXN1	GTG​CCA​CCC​AAC​ATT​AAC​ATC​A	ACC​ACT​TTC​CTC​GCA​TTC​TCT​A
SLC38A2	TAA​TCT​GAG​CAA​TGC​GAT​TGT​GG	AGA​TGG​ACG​GAG​TAT​AGC​GAA​AA
SLC38A1	CCT​TCA​CAA​GTA​CCA​GAG​CAC	GGC​CAG​CTC​AAA​TAA​CGA​TGA​T
SLC6A14	GAC​AGC​TTC​ATC​CGA​GAA​CTT​C	ATT​GCC​CAA​TCC​CAC​TGC​AT
SLC1A5	CAT​CAA​CGA​CTC​TGT​TGT​AGA​CC	CGC​TGG​ATA​CAG​GAT​TGC​GG
β-Actin	ACC​ACA​CCT​TCT​ACA​ATG​AG	ACGACCAGAGGCATACAG

### 2.8 Serum biochemical analysis

The levels of creatinine, urea, AST, and ALT in mouse serum were strictly measured according to the instructions of Elabscience kit.

### 2.9 Metabolomic analysis of serum and tumor samples

For sample preparation, 200 μL methanol containing ^13^C-glutamine (internal standard) was added to 50 μL mouse serum or tumor homogenate for protein precipitation. The supernatant after centrifugation was concentrated and evaporated under vacuum, followed by reconstitution in 200 μL ultrapure water for subsequent analysis. Metabolites were eluted onto an XBridge® Amide column (3.5 μm × 4.6 mm × 100 mm, Waters) and separated using a gradient elution program on a Shimadzu UFLC-30A system (Shimadzu, Kyoto, Japan). The aqueous mobile phase consisted of H2O:ACN 19:1 (v/v), containing 0.1% ammonium acetate (5 mM) and 0.015% ammonia. The organic mobile phase was acetonitrile. Metabolome analysis was performed using ESI in negative ion mode on an AB SCIEX 5600 Q-TOF MS system. The mass spectrometer operated under the following conditions: Gas1, 33 psi; Gas 2, 33 psi; Curtain Gas, 25 psi; ionic atomization voltage, −4500 V; ion source temperature 550 °C; TOF MS1 scan, 50–1000 *m/z*; IDA scanning, 50–900 *m/z*; CE, −20 V; CES, 10; DP, −93 V. Metabolite identification was facilitated using the MASSBANK, METLIN, and MS2T databases. Quantitative analysis was performed using MultiQuant 3.0 software, and multivariate data analysis and modeling were carried out using Metaboanalyst.

### 2.10 Quantitative analysis of amino acids

Serum or tissue samples were processed according to previous report (Shen et al., 2021). Briefly, 500 μL ice-cold acetonitrile, containing 200 ng of 2,5-dihydroxy benzoic acid (DHB) as internal standard, was incorporated into serum or tumor homogenate. Following vacuum drying, 50 μL borate buffer and 50 μL benzoyl chloride were added for the derivatization process. Chromatographic separation was executed on an XBridge® Amide column (3.5 µm × 4.6 mm × 100 mm, Waters). The mobile phase consisted of solvent A (0.2% formic acid and 5.0 mM ammonium formate in water) and solvent B (acetonitrile). MS analysis was performed in the positive mode using a 6500 triple quadrupole mass spectrometer equipped with a turbo ion spray ionization source (Applied Biosystems, Forster City, CA, United States). The MRM conditions for each analyte were outlined in Table 2.

**TABLE 2:** Quantitative parameters of AAs on LC-MS/MS.

Analyte	Precursor *m/z*	Q1-pre deviation (V)	Product *m/z*	Q3-pre deviation (V)	Dwell time (msec)	Collision energy (V)
DHB	380	15	105	15	30	10
Ala	194	15	105	15	30	3
Arg	279	15	105	28	30	15
Asn	237	25	105	10	30	21
Asp	238	15	105	15	30	15
Gln	251	27	105	10	30	5
Leu	236	15	105	15	30	5
Lys	355	17	188	12	30	10
Met	254	27	105	10	30	15
Phe	270	28	120	11	30	10
Pro	220	15	105	15	30	5
Ser	210	23	105	10	30	16
Thr	224	24	105	10	30	7
Tyr	390	15	105	15	30	10
Val	222	15	105	15	30	5
Gly	180	15	105	15	30	5
Glu	252	27	105	19	30	21
Trp	309	15	263	12	30	10

Cells were added to a 12-well plate at 7 × 10^5^ cells/well. After 12 h, LPs at low dose (0.5%) and high dose (1%) were administered to the medium and then cultured for 24 h. The cells and culture supernatants were separately sampled and processed for quantitative analysis.

### 2.11 *In vitro* gut microbiota incubation

Frozen fecal slurry stored at −80 °C it was thawed at 37 °C and diluted 1:5 (v/v) in sterile MRS broth. After 10 min setting, supernatants were collected into sterile 15 mL tubes, centrifuge at 2000 *g* for 2 min, and the pellets resuspended. The OD600 nm was adjusted to around 1.1 with medium and incubated with anaerobic bags at 37 °C for 12 h. For fecal bacteria and drug incubation, pellets were resuspended in 7 mL medium. In 12-well plates, 0.9 mL fecal culture was mixed with 0.1 mL PBS containing LPs (1 mg/mL) or DDP (5 μg/mL). Cultures were incubated anaerobically at 37 °C, and 100 µL aliquots were collected at 0 h, 12 h, and 24 h and quenched with 500 µL ice-cold acetonitrile to stop the reaction, mixed well and stored at −80 °C.

### 2.12 Western blotting (WB)

Protein levels of SFXN1 in tumor tissues were assessed by WB analysis. Equal loading of proteins was verified by α-tubulin. Primary antibodies against SFXN1 and α-tubulin were diluted at 1:1000. The Gel-Pro analyzer software was used for the semi-quantitative analysis of the proteins obtained from the gel imaging system.

### 2.13 Statistical analysis

Multiple comparisons were conducted using Tukey’s multiple comparison test, and others were by one-way ANOVA using Graph Pad Prism 7. The results were shown with mean ± SEM of seven to eight mice per group. Statistically significant difference was considered for *P* < 0.05.

## 3 Results

### 3.1 Antitumor and adjuvant activity of LPs in treating NSCLC

**FIGURE 1:**
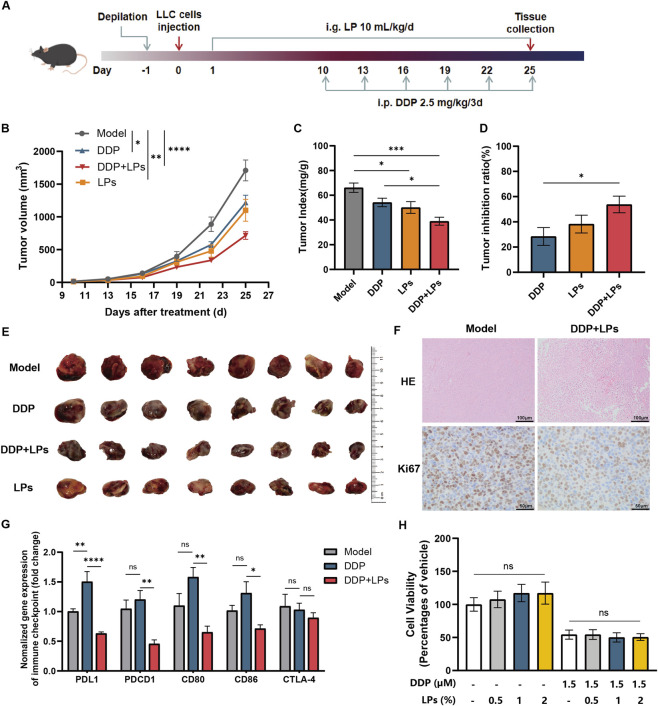
Lienal peptides oral solution enhances cisplatin anti-NSCLC efficacy. **(A)** Mice were orally administrated with LPs (10 mL/kg, everyday) with or without DDP (2.5 mg/kg, every 3 days). n = 8. **(B)** Mouse tumor volume curve during treatment. **(C)** Tumor index (tumor weight/mouse weight). **(D)** Tumor inhibition ratio of mice with administration. **(E)** Pictures of tumor stripped from mice. **(F)** Representative photomicrographs of H&E staining (400×) and Ki67 staining (400×) of the tumor tissue in Lewis tumor-bearing mice. Scare = 100 μm in H&E staining. Scare = 40 μm in Ki67 staining. **(G)** The relative mRNA expression of immune checkpoint PD-L1/PDCD1 and CD80/CD86/CTLA-4 of Lewis tumor-bearing mice (n = 6). **(H)** The relative cell viability of LLC after incubation for 24 h. *P < 0.05, **P < 0.01, ***P < 0.001, ****P < 0.0001.

To examine the effect of adjuvant administration of LPs on the antitumor efficacy of DDP against NSCLC, mice in different groups were respectively administered with LPs, DDP, or DDP + LPs combination, and tumor volumes were measured every 2 days (Figure 1A). Clearly, both DDP and LPs monotherapy reduced the tumor volume and tumor-to-weight ratio relative to the model mice (Figures 1B,C). The combined administration of DDP and LPs produced the smallest tumors and the greatest tumor growth inhibition among all groups (Figures 1D,E), indicating that oral (i.g) LPs not only exhibit standalone antitumor activity but also potentiate DDP. The effect of LPs on the antitumor effect of DDP in tumor tissues were histologically examined using H&E and Ki67 staining. As shown in Figure 1F, the tumor tissues in model group were observed with normal morphology and vigorous cell growth. After the intervention of DDP and LPs, nuclear cleavages significantly reduced, and large areas of cell necrosis and apoptosis, accompanied by hemorrhage were observed. It suggested that DDP co-administered with LPs inhibited tumor proliferation and promoted intratumoral cell apoptosis *in vivo*.

Subsequently, we investigated the effect of LPs on intratumoral immune checkpoints (Figure 1G). Compared with the model and DDP groups, the expression of tumor immune checkpoint PD-L1&PDCD1 in the DDP + LPs group was significantly downregulated. The expression levels of CD80 and CD86 in tumor tissues also showed the same trend, with no significant differences observed in CTLA-4 expression among groups. These findings preliminarily indicated that LPs combined with DDP could significantly inhibit immune evasion in NSCLC mice, which exerted an auxiliary safeguard role for the anti-tumor effect of DDP, and effectively suppressed tumor growth *in vivo*.

To determine the effect of LPs on the tumor cell-killing effect of DDP *in vitro*, we incubated LLC, A549 human NSCLC cells, and HepG2 human hepatocellular carcinoma cells with 1.5 µM DDP and different doses of LPs. The results showed that cancer cells were significantly inhibited by DDP, but LPs had no significant effect on these cells (Figure 1H; Supplementary Figure S1A,B). Similarly, Mouse mononuclear macrophages cells (RAW 264.7) were incubated with 2% LPs combined with DDP. Results showed a significant decrease in cell activity with increasing concentration of DDP, and no improvement was found after LPs with intervention (Supplementary Figure S1C). Therefore, the anti-tumor effect of LPs and its adjuvant anti-tumor effect on DDP were not achieved through direct killing of cancer cells.

### 3.2 Detoxification effect of LPs on DDP

**FIGURE 2:**
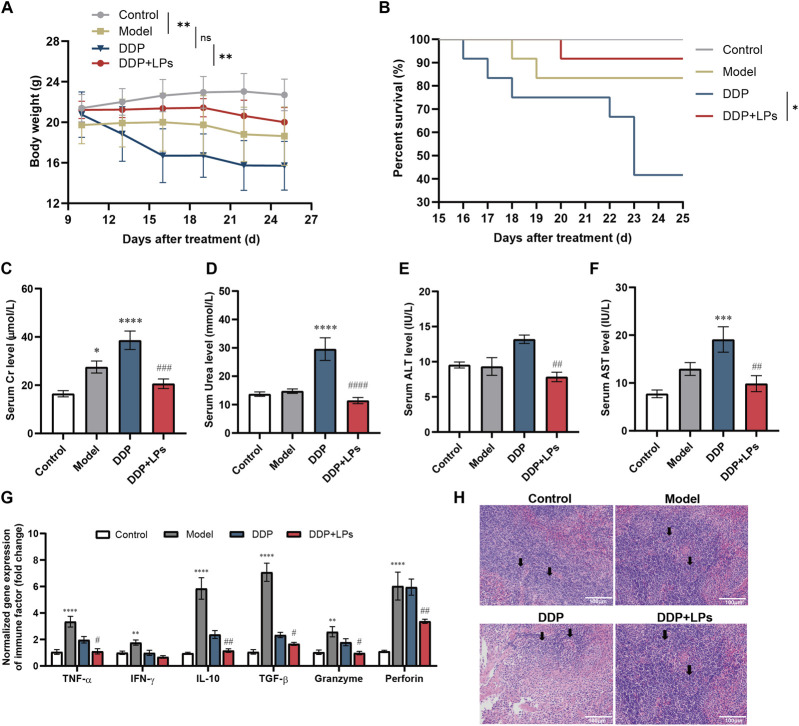
Regulation of cisplatin-induced hepatic and renal injury and splenic immune disorders by lienal peptides oral solution. **(A)** Weight curves and **(B)** Survival curves of Lewis tumor-bearing mice (n = 12). **(C,D)** Serum creatinine and urea concentrations in mice (n = 6). **(E,F)** Serum AST and ALT level in mice (n = 6). **(G)** Relative mRNA level of immune factor TNF-α, IFN-γ, IL-10, TGF-β, Granzyme and Perforin in the spleen of mice (n = 6). **(H)** Representative photomicrographs of H&E staining (200×) of the spleen tissue in Lewis-bearing mice. Scare = 100 μm. RP, Red pulp. The black circles are marked with splenoids and periarterial lymphatic sheaths. *P < 0.05, **P < 0.01, ***P < 0.001, ****P < 0.0001 vs. Con group; ^#^P < 0.05, ^##^P < 0.01, ^###^P < 0.001, ^####^P < 0.0001 vs. DDP group.

Given DDP’s dose-limiting toxicities, we assessed whether LPs mitigate systemic injury at a therapeutic dose of DDP (2.5 mg/kg). After 15 days of DDP treatment, DDP caused progressive weight loss, whereas DDP + LPs maintained body weight above 20 g, indicating a significant difference in average weight between two groups (Figure 2A). According to the Kaplan Meier curve, the survival rate of DDP treated mice was only 50%, with a moderate survival rate of 23 days (Figure 2B). As expected, mice treated in combination with LPs improved survival to 90% and produced a visibly healthier phenotype. Therefore, LPs administration can significantly prolong the survival period and maintain body weight of LLC-bearing mice treated with DDP.

To investigate whether LPs could ameliorate DDP-induced liver and kidney injuries, we examined liver and kidney physiological indexes in the serum of LLC-bearing mice. The results indicated that DDP increased creatinine and urea, while LPs co-administration significantly reversed these elevations (Figures 2C,D). Besides, DDP raised AST and ALT levels relative to the model, consistent with hepatocellular injury, and LPs reduced both markers (Figures 2E,F). These results indicated that LPs combined administration could significantly improve survival and attenuate DDP-induced hepatorenal toxicity.

Tumor burden and chemotherapy also perturb immune homeostasis. Therefore, we then detected the expression of immune factors in the spleens of each group of mice. Obviously, compared with normal mice, the expression levels of immune-promoting factors (TNF-α and IFN-γ), immunosuppressive factors (IL-10 and TGF-β), and killer immune factors (Granzyme and Perforin) were significantly elevated in Lewis tumor-bearing mice (Figure 2G). The co-administration of LPs with DDP significantly downregulated the expression of all immune factors toward baseline compared to the DDP group. Consistently, representative photomicrographs of H&E staining of the spleen tissue showed that the number of lymphocytes in periarterial lymphoid sheath and splenic corpuscle decreased in the DDP-dosed group, and the number of splenic lymphocytes was significantly upregulated by LPs treatment (Figure 2H). Thus, LPs could alleviate the spleen immune suppression and tissue damage caused by DDP administration in LLC-bearing mice.

To further confirm the improvement effect of LPs on DDP’s toxicity, we established a high-dose DDP induced acute liver and kidney injury model. In this process, mice were given 5 mg/kg/3d of DDP after LPs pre-administration for 2 weeks (Supplementary Figure S1D). As expected, LPs administration could not only avoid weight loss caused by DDP (Supplementary Figure S1E), but also significantly downregulated the serum levels of creatinine, urea, ALT, and AST in mice with acute hepatorenal injury (Supplementary Figure S1F-I).

### 3.3 Regulation of metabolomic disturbances in NSCLC mice by LPs

**FIGURE 3:**
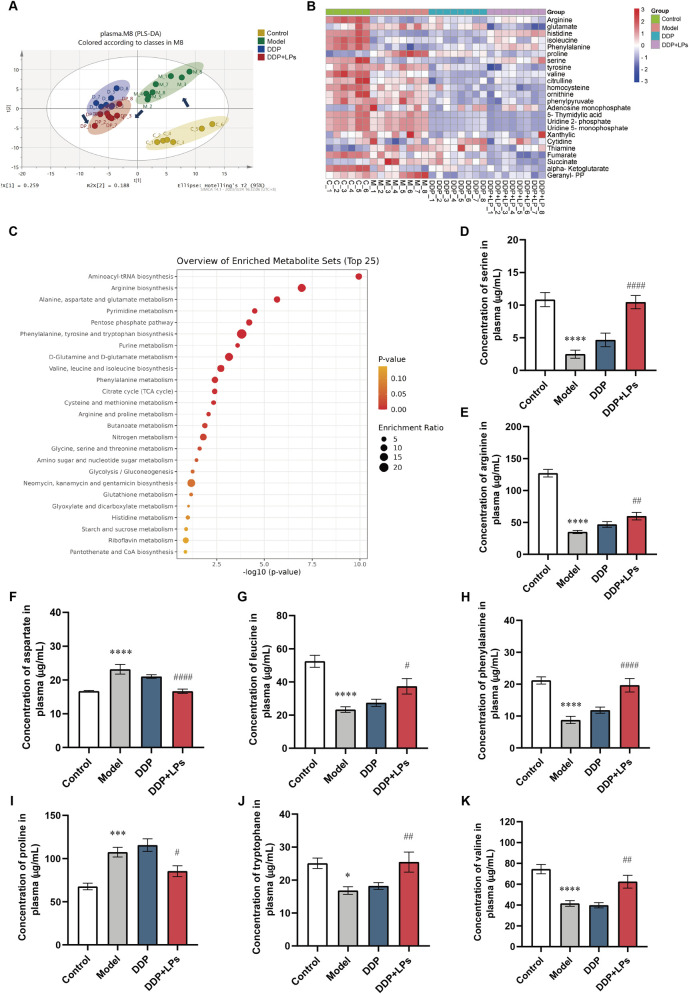
Regulation of serum metabolic profiles and metabolic pathways in Lewis tumor-bearing mice by lienal peptides oral solution combined with cisplatin. **(A)** PLS-DA score plots of serum metabolites in mice. **(B)** Heatmap of serum mebabolites in mice. **(C)** KEGG pathway analysis of serum metabolites. **(D–K)** Concentration of AAs in serum of normal and Lewis bearing-tumor mice (n = 8). *P < 0.05, **P < 0.01, ***P < 0.001, ****P < 0.0001 vs. Con group; ^#^ *P* < 0.05, ^##^ *P* < 0.01, ^###^ *P* < 0.001, ^####^ *P* < 0.0001 vs. Model group.

Given that small molecule metabolism is an important indicator of body homeostasis, we next investigated the regulatory effect of LPs combined with DDP on serum metabolic disorders in LLC-bearing mice to elucidate the detoxification effect of LPs on DDP treatment. Compared with the DDP group, the metabolomic profiles of DDP + LPs group was closer to that of control group (Figure 3A). The heatmap showed that amino acids, organic acids, purines, pyrimidines, nucleotides, and other small molecular metabolites changed dramatically among different groups (Figure 3B). KEGG pathway enrichment assay of differential metabolites highlighted multiple amino acid-related pathways among the 25 most significantly enriched hits (Figure 3C). Subsequently, we carried out absolute quantitative analysis of amino acids in mouse serum of different groups. The results demonstrated that NSCLC modeling led to a significant decrease in levels of serine, arginine, leucine, phenylalanine, tryptophane, valine, in mice, as well as accumulation of aspartate and proline (Figures 3D–K). Wherein, serine had the most pronounced change. The serum serine levels in the model and DDP-dosed mice were significantly lower than those in conventional mice, and administration of LPs significantly upregulated serine levels. Thus, LPs significantly regulated amino-acid dysregulation in NSCLC mice, with serine emerging as a key node.

### 3.4 Hepatorenal protective effects of exogenous serine on LLC-bearing mice

**FIGURE 4:**
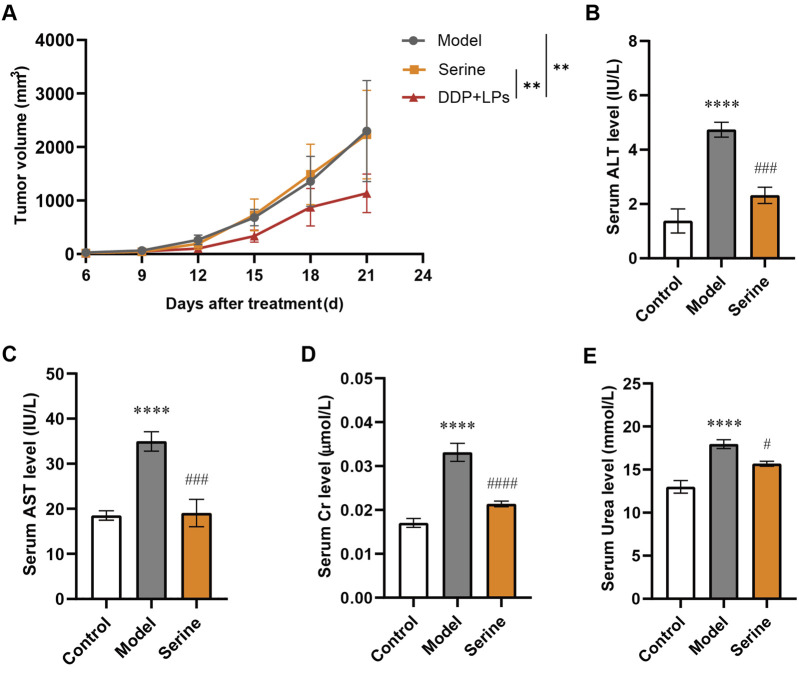
Effect of exogenous serine on Lewis bearing-tumor mice (n = 8). **(A)** Tumor volumes of mice in Control, Model, Serine and DDP + LPs groups (n = 8). **(B,C)** Serum AST and ALT level in mice; **(D,E)** Serum creatinine and urea concentrations in mice. *P < 0.05, **P < 0.01, ***P < 0.001, ****P < 0.0001 vs. Con group; ^#^ *P* < 0.05, ^##^ *P* < 0.01, ^###^ *P* < 0.001, ^####^ *P* < 0.0001 vs. Model group.

In order to obtain further evidence to support the potential detoxification effect of LPs on DDP by regulating the level of serine in the body, we administered 400 mg/kg serine orally for three consecutive weeks in LLC-bearing mice. In the first step, we investigated the anti-tumor effect of serine, and found that exogenous serine did not significantly inhibit tumor growth (Figure 4A). However, the ALT and AST levels in LLC-bearing mice were significantly higher than those of controls, and serine administration could significantly downregulate the levels of serum ALT and AST (Figures 4B,C). Additionally, LLC-bearing mice had higher creatinine and urea levels than those of controls, and serine administration could significantly reduce creatinine and urea levels caused by NSCLC modeling (Figures 4D,E). These data further suggest that increasing peripheral serine exposure may be the mechanism by which LPs reduces the toxicity of DDP in treating NSCLC, but not related to the synergistic effect of LPs on DDP.

### 3.5 The mechanism of LPs up-regulating serum serine exposure

**FIGURE 5:**
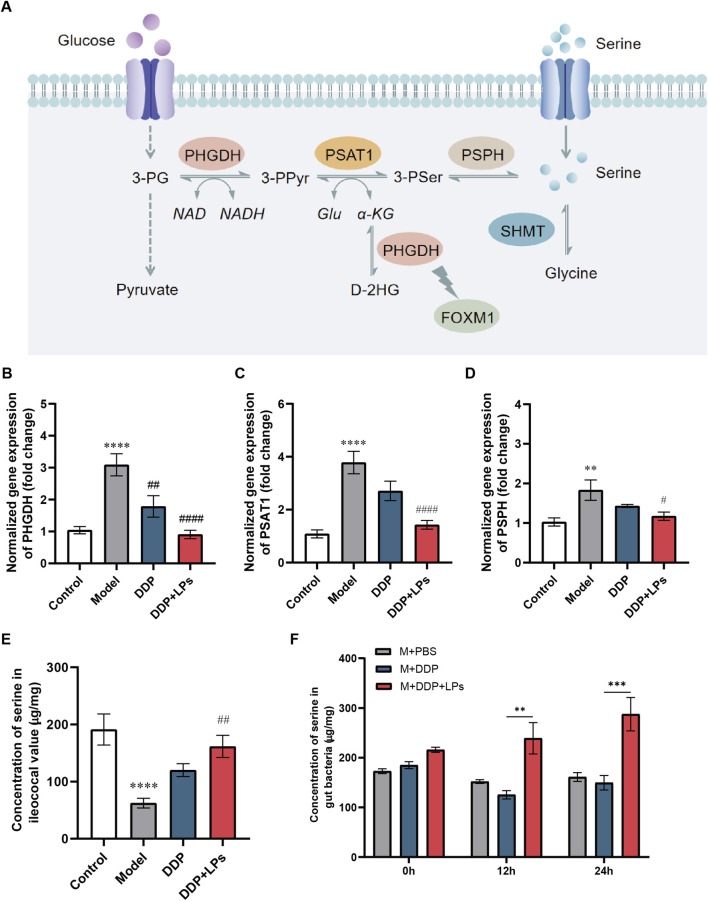
Effect of lienal peptides oral solution combined with cisplatin on serine production in the liver and intestinal flora of Lewis tumor-bearing mice. **(A)** Related compounds and proteins in serine synthesis and transient pathway. **(B–D)** Relative mRNA level of enzyme in serine synthesis pathway (SSP) in mice liver. **(E)** Concentration of AAs in ileococal value of control and Lewis bearing-tumor mice and **(F)** gut bacteria of co-culture with DDP + LPs or DDP. *P < 0.05, **P < 0.01, ***P < 0.001, ****P < 0.0001 vs. Con group; ^#^ *P* < 0.05, ^##^ *P* < 0.01, ^###^ *P* < 0.001, ^####^ *P* < 0.0001 vs. Model group.

It has been found that serine production, metabolic enzymes and transporters are tightly linked to tumor proliferation and growth. The expression of synthase PHGDH (Reid et al., 2018; Lin et al., 2022; Zafeiriadou et al., 2022), PSAT1 (Yang et al., 2015; Guo et al., 2021) and metabolic enzyme SHMT2 (Zeng et al., 2021) are frequently upregulated in tumor tissues of patients with NSCLC and associate with poor prognosis (Figure 5A). Systemically, the main synthesis and decomposition site of serine is located in the liver, yet hepatic serine pathways can be dysregulated in disease (Zhang et al., 2021b). Thus, we analyzed the mRNA levels of PHGDH, PSAT1, and PSPH in liver. Contrary to expectations, the expression of PHGDH, PSAT1, and PSPH was significantly upregulated in the liver of LLC-bearing mice, while LPs administration significantly downregulated the expression of these key serine synthetases (Figures 5B–D), indicating that LPs do not raise serum serine by stimulating hepatic *de novo* synthesis.

Numerous literatures have reported that gut microbiota is an important site for the production of various amino acids. As shown in Figure 5E, LLC-bearing mice had markedly reduced serine exposure relative to conventional controls, which was significantly restored by DDP + LPs. It suggested that the upregulation of peripheral serine exposure by LPs might be related to the regulation of serine production mediated by gut microbiota. Additionally, the gut microbiota of LLC-bearing mice was incubated with DDP or DDP + LPs for 0, 12 and 24 h, respectively. Clearly, DDP alone did not alter serine production, whereas DDP + LPs increased microbial serine generation at 12 h (Figure 5F). When the incubation time was extended to 24 h, the serine production increase in gut microbiota by DDP + LPs was more significant. Together, these data suggest that LPs elevate circulating serine primarily by enhancing microbiota-mediated production.

### 3.6 LPs regulated intratumoral serine and transport pathways

**FIGURE 6:**
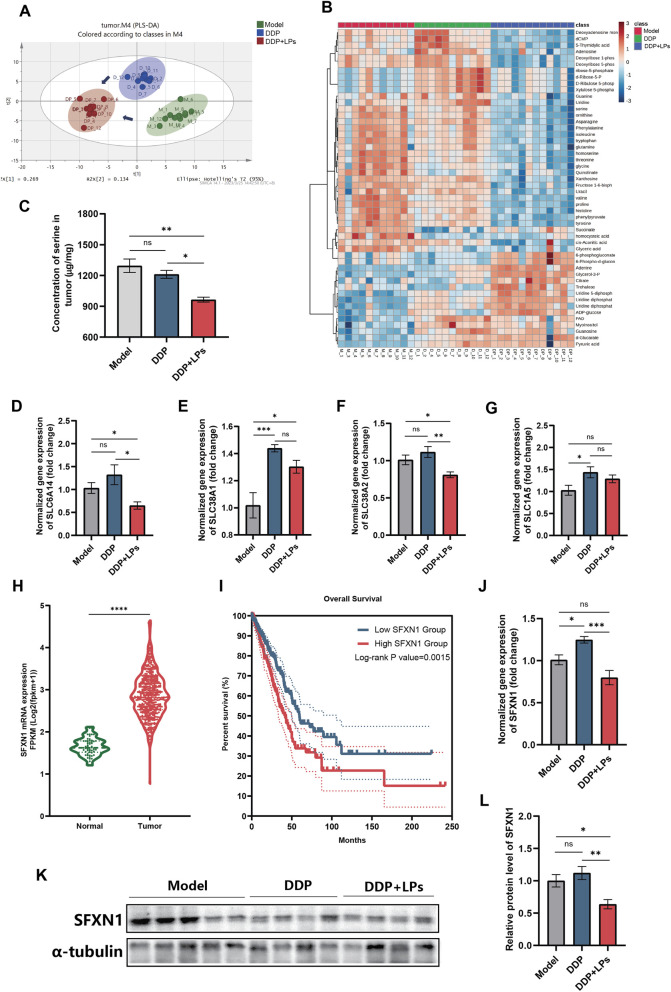
Analysis of amino acid profile and SFXN1 expression in Lewis tumor-bearing mice by lienal peptides oral solution combined with cisplatin. **(A)** Heatmap of tumor metabolome. **(B)** PLS-DA score plots of serum metabolites in mice. **(C)** Concentration of serine in tumor of Lewis bearing-tumor mice (n = 8). **(D–G)** Relative mRNA level of neutral amino acid transporter in tumor. **(H)** SFXN1 gene expression in lung adenocarcinoma tumor and normal tissue. **(I)** Overall survival of lung adenocarcinoma tumor patient with different SFXN1 gene expression level. **(J)** Relative SFXN1 mRNA expression and **(K,L)** protein level in tumor tissue. *P < 0.05, **P < 0.01, ***P < 0.001, ****P < 0.0001.

Metabolomics technique was used to analyze the differences in intratumoral small molecule metabolites among different groups. As shown in Figure 6A, there was a significant difference in metabolites within tumors among three groups. It was found in KEGG analysis that purine metabolism, pyrimidine metabolism, aminoacyl-tRNA biosynthesis, alanine, aspartate and glutamate metabolism pathways showed the most significant changes (Supplementary Figure S2A,B). The heatmap also revealed broad shifts in amino acids, organic acids, purines, pyrimidines, and nucleotides (Figure 6B). Next, we carried out absolute quantitative analysis of amino acids in tumor. Obviously, DDP + LPs treatment significantly reduced the exposure of serine in the tumors of LLC-bearing mice (Figure 6C), along with histidine, proline, valine, 5-thymidylic acid and deoxyribose 1′-phosphate (Supplementary Figure S2C,2I), while increasing glycerol-3′-phosphate. The opposite trend of serine in serum and tumor suggested that the increase of serine in serum by LPs might also be related to altered tumor uptake or transport.

The rapid proliferation of tumors requires amino acids. Therefore, we hypothesized that LPs can inhibit the expression of amino acid uptake transporters on tumors, thereby increasing the level of peripheral circulating amino acids. To confirm this hypothesis, we first quantitatively detected the expression of neutral amino acid transporters in tumors, including SLC6A14, SLC1A1, SLC38A1, SLC38A2, and SLC1A5. As shown in Figures 6D–G, compared with the model group and DDP treatment group, DDP + LPs treatment could downregulate the expression of SLC6A14 and SLC38A2, while the mRNA level of SLC38A1 in the DDP + LPs group was significantly upregulated compared to the model group, with no impact on SLC1A5. Thus, the decrease of intratumoral amino acid levels caused by DDP + LPs administration might be related to the downregulation of SLC6A14 and SLC38A2.

According to previous reports, SFXN1, as a mitochondrial transport protein of serine, forms a carbon unit and is a key transport protein for cell growth (Kory et al., 2018). We thus analyzed the difference of SFXN1 expression between lung adenocarcinoma and normal tissues and the relationship between SFXN1 expression and survival time of lung adenocarcinoma patients by using GDC TCGA LUAD database on UCSC Xena platform. As illustrated in Figures 6H,I, compared with normal lung tissue, SFXN1 was highly expressed in lung adenocarcinoma, and the overall survival time of lung adenocarcinoma patients with high SFXN1 expression was significantly shorter than those with low SFXN1 expression. We then analyzed the mRNA and protein expression of SFXN1 in the tumor tissues of LLC-bearing mice. The result showed that mRNA level of SFXN1 of DDP + LPs group was significantly lower than that of model and DDP-dosed groups (Figure 6J). The WB analysis results also demonstrated that the combination of LPs and DDP significantly downregulated the expression of SFXN1 in the tumor of LLC-bearing mice (Figures 6K,L). At the cellular level, mRNA level of SFXN1 in LLC cells was reduced upon LPs administration (Supplementary Figure S3A). In line with this notion, the concentrations of serine in cells and medium were analyzed. The results demonstrated that LPs might inhibit the transport of serine into LLC cells (Supplementary Figure S3B,C).

## 4 Discussion

Protein hydrolysates have been recognized as effective sources of bioactive peptides with diverse functionalities such as antithrombotic, antihypertensive, antimicrobial, anticancer, and antioxidant properties, along with various immunomodulatory effects (Kiewiet et al., 2018). The peptides, with molecular weights falling precisely between those of whole proteins and small molecules, have emerged as promising agents. As specific candidate drugs, their degradation products naturally participate in cellular nutrient supply and cell construction, implying that peptides might present a more synergistic regulation of immunity *in vivo* than that achieved by hormone agents. With the ongoing discovery of numerous protein/peptide receptors and protein-related signaling pathways, tumor-targeted peptides hold promising prospects (Pan et al., 2020). LPs, extracted from healthy porcine spleen, have been reported as unique immune regulators that alleviate immunosuppression caused by cyclophosphamide and may benefit cancer treatment. Nevertheless, the safety and efficacy of LPs, alone or in combination with chemotherapy for cancer treatment, still require preclinical and clinical validation (Huang et al., 2015).

In the present study, we investigated the potential of spleen peptides derived from porcine combined with DDP as an adjuvant therapy for NSCLC *in vivo* and *in vitro*. Our results demonstrated that LPs combined with DDP could significantly inhibit immune evasion in NSCLC mice, which exerted an auxiliary safeguard role for the anti-tumor effect of DDP, and effectively suppressed tumor growth in LLC-bearing mice. More importantly, LPs could not only promote the anti-tumor effect of DDP, but also exert anti-tumor effects alone. However, DDP + LPs nither further inhibited tumor proliferation nor enhanced the cytotoxic effects of DDP on normal cells and immune cells, indicating that the adjuvant benefit is unlikely to stem from direct cancer-cell killing. Although certain cationic peptides can fold into membrane-active structures that lyse cancer cells (Sinthuvanich et al., 2012), our data suggest that LPs act predominantly through indirect host-mediated mechanisms. Our future research will further investigate the anti-tumor mechanism of LPs based on indirect mechanisms such as microbial community regulation and intestinal epithelial immune regulation.

In terms of detoxification effects, survival analysis demonstrated that DDP + LPs prolonged the survival time of mice and mitigated the weight loss induced by DDP. Additionally, LPs alleviated the hepatorenal injury aggravated by DDP, and the expression of spleen immune factors and spleen pathological sections suggested that LPs could rectify DDP-related immune disorder and spleen function damage, consistent with previous reports on bioactive peptides (Xie et al., 2002). Some types of protein hydrolysates can stimulate reactive oxygen species (ROS), which triggers the nonspecific immune defense system and inhibits the proliferation of spleen lymphocytes in mice (Sánchez and Vázquez, 2017). Herein, metabolomics technique was used to further explore potential targets for LPs to exert detoxification effects on DDP. Our results showed that LPs administration significantly corrected DDP-exacerbated metabolic disturbances in NSCLC mice, with serine showing the most significant change. Subsequently, absolute quantification of amino acids in normal mice and LLC-bearing mice were conducted using LC-MS/MS. The serum serine levels in the model and DDP-dosed mice were significantly lower than those in conventional mice, and DDP + LPs treatment significantly upregulated serine levels. Thus, we speculated that serine might be a targeted small molecule metabolite for the combined adjuvant therapy of LPs and DDP in NSCLC. According to the literature, serine plays a pivotal role in tumor formation and evolution (Amelio et al., 2014). To investigate whether LPs exert its detoxification effect on DDP by regulating the systemic level of serine, we administered 400 mg/kg of exogenous serine by gavage for three consecutive weeks in LLC-bearing mice. By supplementing exogenous serine, we observed an improvement in liver and kidney injury in LLC-bearing mice, though it had no effect on tumor growth. Literature on enteric-derived serine aligns with this renoprotective signal and links intestinal serine production to protection against hepatorenal injury (Nakade et al., 2018). Together, these observations suggest that increased peripheral serine exposure during LPs administration may be the molecular mechanism by which LPs reduces the toxicity of DDP in treating NSCLC, but is not related to the synergistic effect of LPs on DDP.

The expression of synthase PHGDH, PSAT1 and metabolic enzyme SHMT2 is abnormally increased in tumor tissues of patients with NSCLC, which is closely related to poor prognosis (Yang et al., 2015; Reid et al., 2018; Guo et al., 2021; Zeng et al., 2021; Lin et al., 2022; Zafeiriadou et al., 2022). To explore the mechanism by which LPs increased serum serine, we investigated the effect of LPs combined with DDP on liver serine synthase (PHGDH, PSAT1 and PSPH). Contrary to expectations, LPs administration significantly downregulated the expression of these key serine synthetases. Thus, the increase in serum serine levels in NSCLC mice by LPs is not achieved by promoting hepatic synthesis. Since numerous literatures have suggested that the gut microbiota can mediate the metabolism of systemic amino acids, we next investigated the regulatory effect of LPs on the production of serine by gut microbiota. *In vitro* incubation experiments showed that DDP alone did not alter microbial serine output, whereas DDP + LPs increased serine production at 12 h and more robustly at 24 h, consistent with *in vivo* recovery of serine exposure at the ileocecal valve. Additionally, the relationship between serine and intestinal flora has been reported in several studies. Clostridia have been investigated to produce serine proteases, which may be relevant to the pathogenesis of inflammation bowel disease (Kriaa et al., 2021). Besides, exogenous administration of serine decreases the concentration of myeloperoxidase, eosinophil peroxidase, and proinflammatory cytokine in colonic tissues. More importantly, results of 16S rRNA showed that exogenous serine altered colony composition of gut microbiota (Zhang et al., 2018).

Finally, we explored why tumor serine exposure fell despite higher systemic serine with DDP + LPs. We observed downregulation of neutral amino-acid transporters SLC6A14 and SLC38A2, suggesting reduced tumor import of circulating amino acids. We also focused on SFXN1, a mitochondrial transport protein of serine that fuels one-carbon metabolism to sustain proliferation (Kory et al., 2018). The overexpression of SFXN1 has been implicated in promoting lung cancer cell progression and inhibiting apoptosis, while its knockdown exhibits the opposite effect. Furthermore, it was found that SFXN1 may synergize with c-Myc/AKT/P70 (S6K) to promote lung cancer progression through mTOR activation (Chen et al., 2022). By using GDC TCGA LUAD database on UCSC Xena platform, we found that the high expression of SFXN1 in lung adenocarcinoma was closely related to the survival of tumor patients. Then, we analyzed the mRNA and protein expression of SFXN1 in mouse tumor tissue, and found that the expression of SFXN1 in NSCLC mouse tumors was downregulated by LPs. This may further reduce the tumor cells’ utilization of serine, disrupt intracellular redox homeostasis, and thereby affect the biological functions of tumor cells such as proliferation, migration and invasion. Moreover, small peptides, once into the body, can cross the intestinal barrier and be detected in the blood of volunteers consuming dairy and soybean products, thereby directly affecting immune cells in systemic circulation (Dia et al., 2009). Furthermore, bioactive peptides can be internalized by PepT1 transporters or be absorbed into cells via liquid-phase endocytosis (Knipp et al., 1997). Once absorbed by epithelial or immune cells, immunomodulatory peptides can interfere with signal pathways (Kiewiet et al., 2018), supporting our hypothesis that LPs exerts its antitumor effects through indirect pathways. Together, these results provide compelling evidence for the mechanism underlying the efficacy of LPs combined with DDP in the treatment of NSCLC.

## 5 Conclusion

Combining LPs with DDP can increase systemic serine exposure, primarily via microbiota-driven serine production, while concurrently restricting intratumoral serine utilization, which may be related to downregulation of SFXN1. This dual action mitigates DDP-induced hepatorenal and immune injury and improves the therapeutic index in NSCLC, supporting LPs as a mechanistically grounded adjuvant to DDP therapy.

## Data Availability

The data presented in the study are deposited in the Mendeley Data repository, DOI: 10.17632/f4dffdt7xf.1.
