# Supplementary material for: Serine synthesis and transport mediate the synergistic and detoxifying effects of lienal peptides on cisplatin
Source: Front Pharmacol. 2026 Jan 16;16:1646217. doi: 10.3389/fphar.2025.1646217 (PMC12855131; doi:10.3389/fphar.2025.1646217)
Supplement: Supplementary file 1 [file DataSheet1.docx]

**Supplementary Figures**


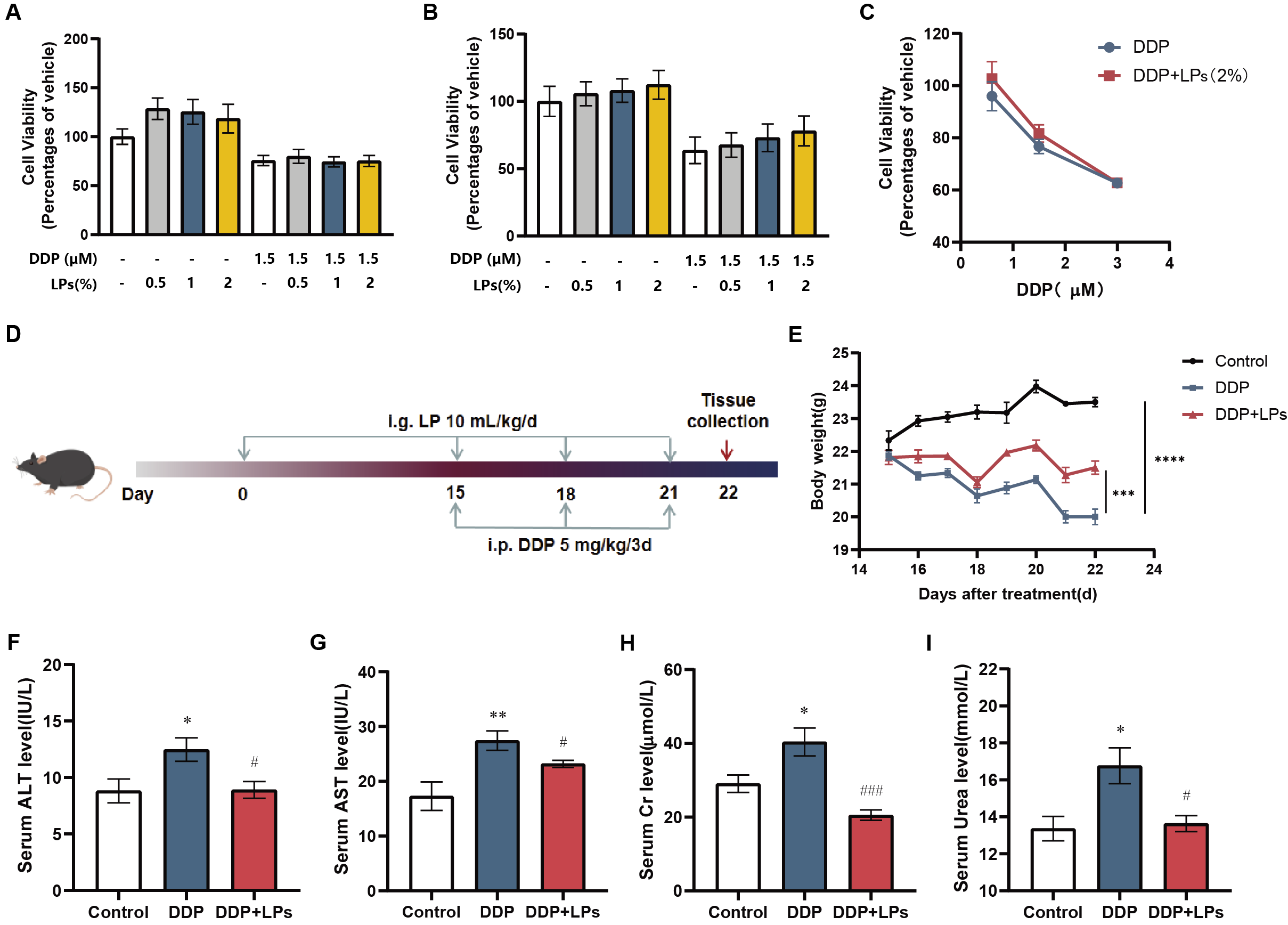


**Fig.S1** Effects of LPs on different cells *in vitro* and acute toxicity of DDP *in vivo.* The relative cell viability of (A) A549, (B) HepG2 and (C) RAW264.7 after incubation for 24h. (D) Healthy mice were orally administrated with LPs (10mL/kg, every three days) with or without DDP (5mg/kg, every three days). n=6. (E) Weight curves of mice in control, DDP and DDP+LP group. (F and G) Serum AST and ALT level in mice.(H and I) Serum creatinine and urea concentrations in mice. **P*<0.05, ***P*<0.01 *vs* Con group; ^#^*P*<0.05, ^##^*P*<0.01,^###^*P*<0.001 *vs* DDP group.


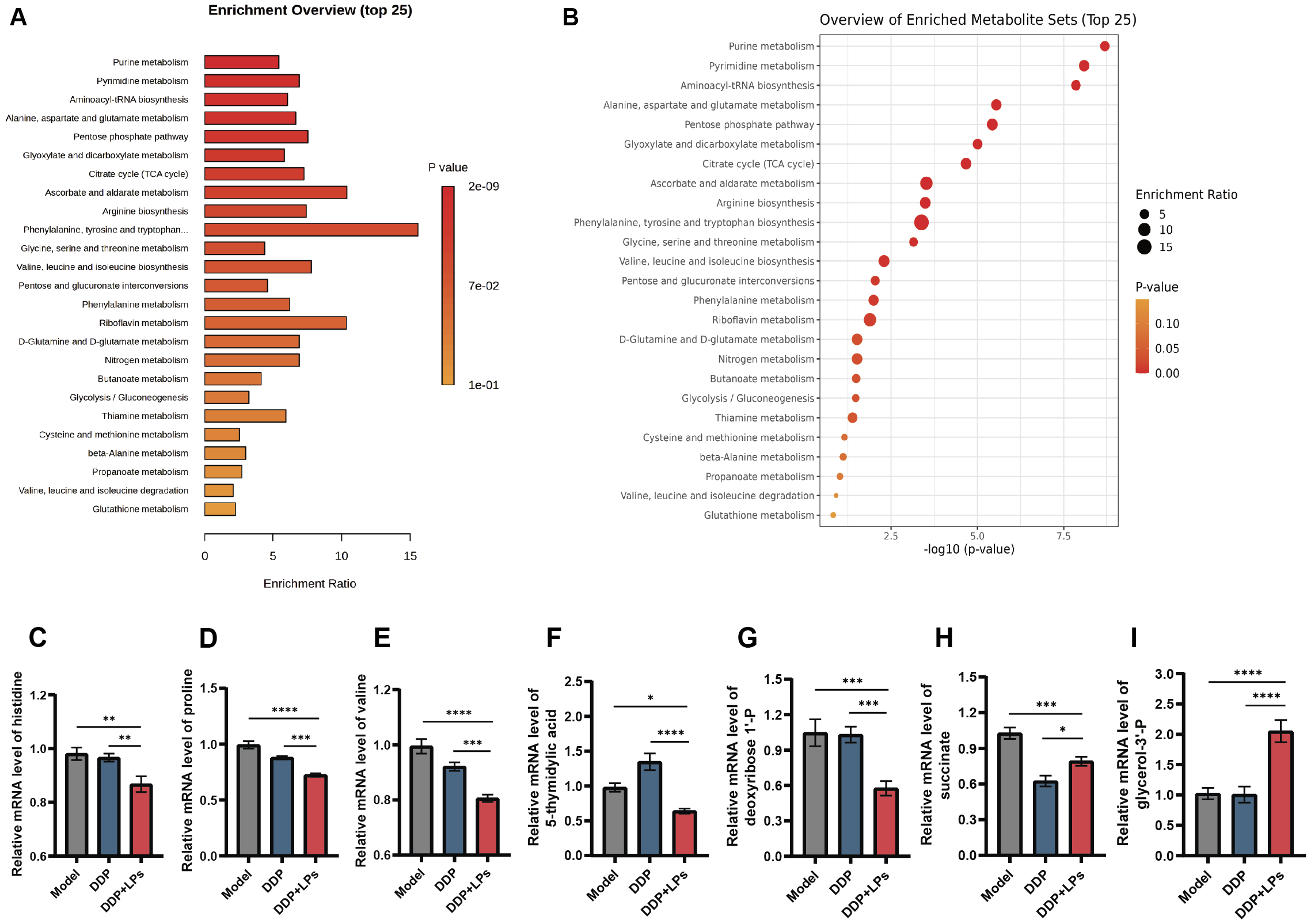


**Fig.S2** Analysis of tumor metabolites in Lewis tumor-bearing mice. (A and B) Pathway analysis of tumor metabolites. (C-I) Relative level of tumor metabolites. **P*<0.05, ***P*<0.01, ****P*<0.001, *****P*<0.0001.


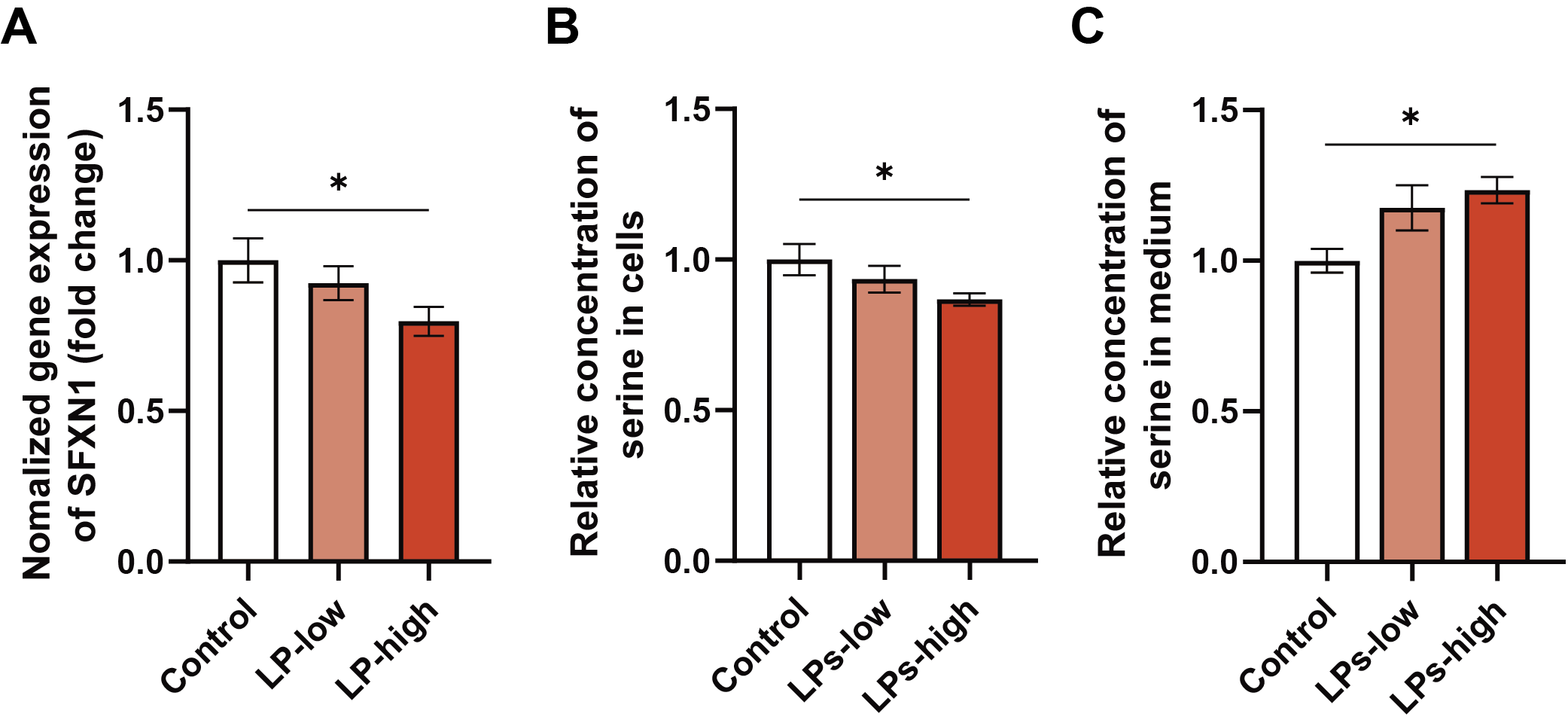


**Fig.S3** The effect of LPs on serine uptake in LLC cells. (A) SFXN1 gene expression in cells after treatment. (B and C) Relative concentration of serine in cells and medium with administration of LPs at low dose (0.5%) and high dose (1%).**P*<0.05.
